# Characterization of Prostate Cancer Bone Metastases According to Expression Levels of Steroidogenic Enzymes and Androgen Receptor Splice Variants

**DOI:** 10.1371/journal.pone.0077407

**Published:** 2013-11-07

**Authors:** Emma Jernberg, Elin Thysell, Erik Bovinder Ylitalo, Stina Rudolfsson, Sead Crnalic, Anders Widmark, Anders Bergh, Pernilla Wikström

**Affiliations:** 1 Department of Medical Biosciences, Pathology, Umeå University, Umeå, Sweden; 2 Department of Surgical and Perioperative Sciences, Orthopedics and Urology and Andrology, Umeå University, Umeå, Sweden; 3 Department of Radiation Sciences, Oncology, Umeå University, Umeå, Sweden; University of Nebraska Medical Center, United States of America

## Abstract

**Background:**

Intra-tumoral steroidogenesis and constitutive androgen receptor (AR) activity have been associated with castration-resistant prostate cancer (CRPC). This study aimed to examine if CRPC bone metastases expressed higher levels of steroid-converting enzymes than untreated bone metastases. Steroidogenic enzyme levels were also analyzed in relation to expression of constitutively active AR variants (AR-Vs) and to clinical and pathological variables.

**Methodology/Principal Findings:**

Untreated, hormone-naïve (HN, n = 9) and CRPC bone metastases samples (n = 45) were obtained from 54 patients at metastasis surgery. Non-malignant and malignant prostate samples were acquired from 13 prostatectomy specimens. Transcript and protein levels were analyzed by real-time RT-PCR, immunohistochemistry and immunoblotting. No differences in steroidogenic enzyme levels were detected between CRPC and HN bone metastases. Significantly higher levels of SRD5A1, AKR1C2, AKR1C3, and HSD17B10 mRNA were however found in bone metastases than in non-malignant and/or malignant prostate tissue, while the CYP11A1, CYP17A1, HSD3B2, SRD5A2, and HSD17B6 mRNA levels in metastases were significantly lower. A sub-group of metastases expressed very high levels of AKR1C3, which was not due to gene amplification as examined by copy number variation assay. No association was found between AKR1C3 expression and nuclear AR staining, tumor cell proliferation or patient outcome after metastases surgery. With only one exception, high AR-V protein levels were found in bone metastases with low AKR1C3 levels, while metastases with high AKR1C3 levels primarily contained low AR-V levels, indicating distinct mechanisms behind castration-resistance in individual bone metastases.

**Conclusions/Significance:**

Induced capacity of converting adrenal-gland derived steroids into more potent androgens was indicated in a sub-group of PC bone metastases. This was not associated with CRPC but merely with the advanced stage of metastasis. Sub-groups of bone metastases could be identified according to their expression levels of AKR1C3 and AR-Vs, which might be of relevance for patient response to 2^nd^ line androgen-deprivation therapy.

## Introduction

The first-line treatment for patients with advanced prostate cancer (PC) is androgen deprivation therapy (ADT). This therapy is effective in most patients, but after a period of initial remission tumors generally relapse, predominantly within the bone, and are then termed castration-resistant PC (CRPC).

In spite of low circulating levels of androgens in castrated men the majority of CRPC tumors show androgen receptor (AR) activity and expression of AR regulated genes [1]. Possible mechanisms behind AR activity in CRPC include AR gene amplification and mutations, intra-tumoral synthesis of androgens, and expression of constitutively active AR splice variants [2], [3]. Notably some CRPC tumors, and individual tumor cells in most patients, show very low or absence of nuclear AR immunostaining, and factors down-stream the AR are not necessarily up-regulated in those cases [4], [5], [6], [7]. We have recently characterized a series of hormone-naïve (HN) and CRPC bone metastases in patients according to AR activation and expression of AR splice variants (AR-Vs) [5], [8]. Levels of the AR-V7 (also termed AR3) [9], [10] and AR-V567es [11] variants were increased in CRPC compared to HN metastases and, furthermore, found to be highly expressed in a sub-group of CRPC patients with particularly poor prognosis [8]. The AR-V7 and AR-V567es lack ligand-binding domain (LBD) and possess constitutive activity [9], , and patients expressing those AR-Vs probably show poor response to ADT and anti-AR drugs targeting the LBD. Some CRPC patients however respond to 2^nd^ line ADT and this might be due to intra-tumoral steroidogenesis and production of testosterone, dihydrotestosterone (DHT), or other androgens at levels high enough for AR activation, as reported by others [13], [14], [15], [16]. It is, however, not known when these steroidogenic enzymes are up-regulated. Are they increased already in previously untreated HN metastases, or as the AR-Vs [8] increased as result of castration treatment? One aim of this study was therefore to analyze expression of steroid-converting enzymes potentially involved in synthesis of testosterone and DHT in a set of HN and CRPC bone metastases obtained from patients at metastasis surgery. Furthermore, enzyme expression levels were analyzed in relation to expression of AR-Vs, in order to identify potential different mechanisms behind CRPC in individual bone metastases.

## Materials and Methods

### Ethics statement

The study was approved by the local ethic review board of Umeå University and participants gave written or verbal consent. Due to the acute situation when bone metastasis surgery is performed in order to relief spine symptoms and paresis, logistics do not always allow written consent and the local ethic review board therefore specifically approved also verbal consent. Verbal consent is documented by the physician in the patient journal.

### Patients

Bone metastasis tissue was obtained from series of fresh-frozen and formalin-fixed biopsies collected from patients with PC operated for metastatic spinal cord compression or pathologic fractures at Umeå University Hospital (2003–2011). Clinical characteristics and therapies are summarized in Table 1, and this patient series has been previously described in Crnalic et al [5], [17], [18]. The study also includes 13 patients who were treated with radical prostatectomy at Umeå University Hospital, between Feb 2005 and Sep 2006. Median age for the patients was 60 years (range 48–68 years) and median PSA were 6.6 ng/ml (range 3.5–24 ng/ml). Eleven of the patients had tumors graded as Gleason score (GS) 7 and two as GS 8. Four of the tumors were in stage T2 and nine in stage T3. Additional information about tissue handling has been previously described in Hörnberg et al [8].

**Table 1:** Clinical and histological characteristics of prostate cancer patients with bone metastases and treated with metastasis surgery.

Clinical characteristics	Hormone-naíve	Castration-resistant^a^
	n = 9	n = 45
**Age at PCa diagnosis** (yrs)	77 (60–85)	71 (45–86)
**Age at surgery** (yrs)	¨	73 (54–88)
**Serum PSA at PCa diagnosis** (ng/ml)	172 (21–10000)	60 (2–7300)
**Serum PSA at surgery** (ng/ml)	¨	146 (0–5139)
**Bicalutamide prior to surgery**		
Yes	0	29
No	9	16
**Radiation prior to surgery** b		
Yes	0	5
No	9	40
**Chemotherapy prior to surgery**		
Yes	0	5
No	9	40
**Follow-up after surgery** c (months)	40.4 (19.7–98.7)	5 (0.10–68.7)
**AR score** d	8 (1–12)	8 (0–12)
**PSA score** d	9 (0–12)	6 (0–12)
**Proliferation index** e	14.6 (4.1–31.4)	14.4 (3.5–80.0)

Continuous values are given as median (min-max values).

a Castration-resistant patients had disease progression after long-term androgen deprivation therapy including surgical ablation, LHRH/GNRH agonist therapy, and therapy with anti-androgen (bicalutamide).

b Radiation towards operation site.

c The time between date of surgery and the latest follow-up examination or death.

d Androgen receptor (AR) and prostate specific antigen (PSA) immunohistochemical staining scores were assessed according to details described in the materials and methods section.

e Proliferation indexes were assessed as fractions of proliferating (Ki67 immunostained) tumor epithelial cell, according to details described in the materials and methods section.

### Sample preparation

Representative areas of bone metastases, primary PC and non-malignant prostate tissue were cryo-sectioned into extraction tubes and RNA was isolated using the Trizol protocol (Invitrogen, Stockholm, Sweden) or AllPrep DNA/RNA/Protein Mini Kit (Qiagen, Sollentuna, Sweden). For cases were DNA and protein were analyzed in parallel to RNA, nucleic acids and protein were isolated from the same tissue sections using the AllPrep method. The percentages of tumor cells in the samples were above 50% in the majority of cases. The RNA and DNA concentrations were quantified by absorbance measurements using a spectrophotometer (ND-1000 spectrophotometer; NanoDrop Technologies, Inc.,Wilmington, DE). Protein concentration was determined by the BCA Protein assay (Pierce Chemical Co., IL, USA). The RNA quality was analyzed with the 2100 Bioanalyzer (Agilent Technologies, Santa Clara, CA, USA) and verified to have a RNA integrity number ≥6.

### Real time RT-PCR

RNA was reversed transcribed with Superscript II reverse transcriptase (Invitrogen) in a total volume of 10 µl. The subsequent PCR amplification was performed using the Biorad iQ5 iCycler (Bio-Rad Laboratories, Hercules, CA) or the ABI PRISM 7900HT Instrument (Applied Biosystems, Inc., Foster City, CA) in a total volume of 20 µl using the IQ SYBR Green Supermix (Bio-Rad laboratories) or the TaqMan gene expression mastermix (Applied Biosystems, Life Technologies Europe BV, Stockholm, Sweden) according to manufacturers' protocols. Primer sequences are shown in Table S1. Each sample was run in duplicates and adjusted for the corresponding RPL13A mRNA levels. Melting curve analysis confirmed the amplified products, which were also analyzed by 1% agarose gel electrophoresis to confirm the expected size (data not shown).

### Immunohistochemistry

Samples were fixed in buffered formalin, decalcified in formic acid, and embedded in paraffin [5]. Five µm paraffin sections were deparaffinized according to standard procedures, boiled in 0.01 M citrate buffer, pH 6.0, and immunostained for AKR1C3 (NP6-G6.A6, diluted 1∶1000, Sigma, Saint Louis, Missouri, USA), AR (PG-21, diluted 1∶25, Upstate, Lake Placid, NY, USA), PSA (A0562, diluted 1∶2000, DAKO, Glostrup, Denmark) and Ki67 (MIB1, diluted 1∶50, DAKO) with Ultraview Universal DAB Detection Kit (Ventana Medical Systems). Nuclear AR and cytoplasmatic AKR1C3 and PSA staining in tumor epithelial cells were scored according to intensity (0 = no staining, 1 = weak, 2 = moderate, 3 = intense staining) and fraction of stained cells (1 = 1–25%, 2 = 26–50%, 3 = 51–75%, 4 = 76–100%). A total score (ranging from 0–12) was obtained by multiplying the staining intensity and fraction scores. Ki67 were scored by evaluating 300–1000 tumour epithelial cells per patient, as described earlier [5].

### Copy number analysis

The copy number of the AKR1C3 gene in PC bone metastases was examined using the Hs03060693_cn and the Hs02574521_cn TaqMan copy number assays (Applied Biosystems, Life Technologies Europe BV, Stockholm, Sweden), targeting AKR1C3 exon 2 and exon 7, respectively. The assays were run according to the manufacturer's description, with RNaseP as reference gene, and analyzed using the Copy Caller software (Applied Biosystems).

### Immunoblotting

22Rv1 cells were maintained according to manufacturer's instructions (ATCC) and protein was extracted as above. Samples (10 µg protein) were separated by 7.5% SDS-PAGE under reducing conditions and subsequently transferred to PVDF membranes (Immobilon-P, Millipore, Billerica, MA). Membranes were blocked in 5% milk followed by primary antibody incubation in 4°C over night (N-20 antibody, diluted 1∶500 in 1% milk/PBST, Santa Cruz Biotechnology, Santa Cruz, CA) in order to detect the full length AR and approximately 80 kDa truncated AR-Vs with intact N-terminals. The secondary anti-rabbit IgG (Dako, Glostrup, Denmark) antibody (diluted 1∶20 000 in 2.5% milk) was applied after washing in PBST and incubated for 1 h in RT. Protein expression was visualized after extensive washing using the ECL Advanced detection kit (GE Healthcare, Buckinghamshire, UK). Filters were then stripped, according to standard procedures, and analyzed for actin protein levels using the rabbit anti-actin antibody from SIGMA (Saint Louis. Missouri), diluted 1∶8000, in order to ensure equal sample loading. ECL signals were quantified using a ChemiDoc scanner and the Quantity One 4 software (Bio-Rad Laboratories). The intensity of the 80 kDa band in each patient sample was analyzed and expressed in relation to the corresponding full length AR band intensity. The 22Rv1 cell extract was run as a positive control sample on each gel.

### Statistical analysis

Correlations between variables were analyzed using Spearman rank test. Groups were compared using the Mann-Whitney U test for continuous variables and the Chi-square test for categorical variables. Kaplan-Meier survival analysis was performed with death of PC as event and follow-up time as time between metastasis surgery and the latest follow-up examination (December 2012). Statistical analyses were performed using the Statistical Package for the Social Sciences, SPSS 20.0 software (SPSS, Inc, Chicago, USA). A P-value less or equal to 0.05 was considered statistically significant.

## Results

### No general induction of steroidogenic enzymes in castration-resistant compared to hormone-naïve bone metastases

**Figure 1:**
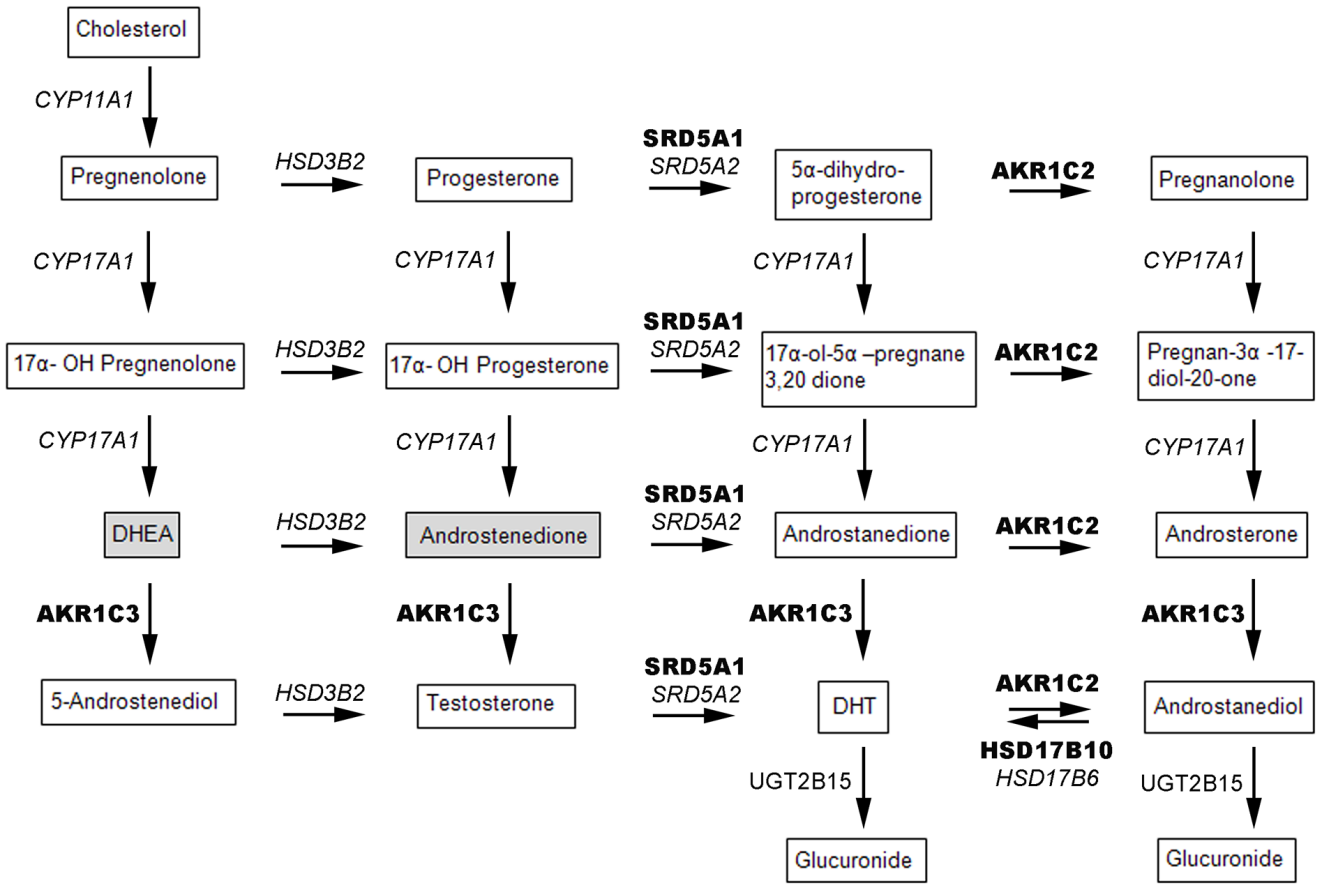
Simplified scheme of the key steps of testosterone and DHT steroidogenesis. Enzymes with significantly higher mRNA levels observed in bone metastases than in non-malignant and/or malignant prostate tissue are shown in bold and enzymes with significantly lower levels observed in bone metastases are shown in *italics*. Steroids secreted by the adrenal gland are highlighted in gray.

Gene expression levels of key enzymes involved in the formation of testosterone and DHT from cholesterol (Figure 1) were assessed by RT-PCR analysis of HN (n = 9) and CRPC bone metastases (n = 45) in comparison with primary peripheral zone prostate tumor (n = 13) and non-malignant prostate tissue (n = 13).

**Figure 2:**
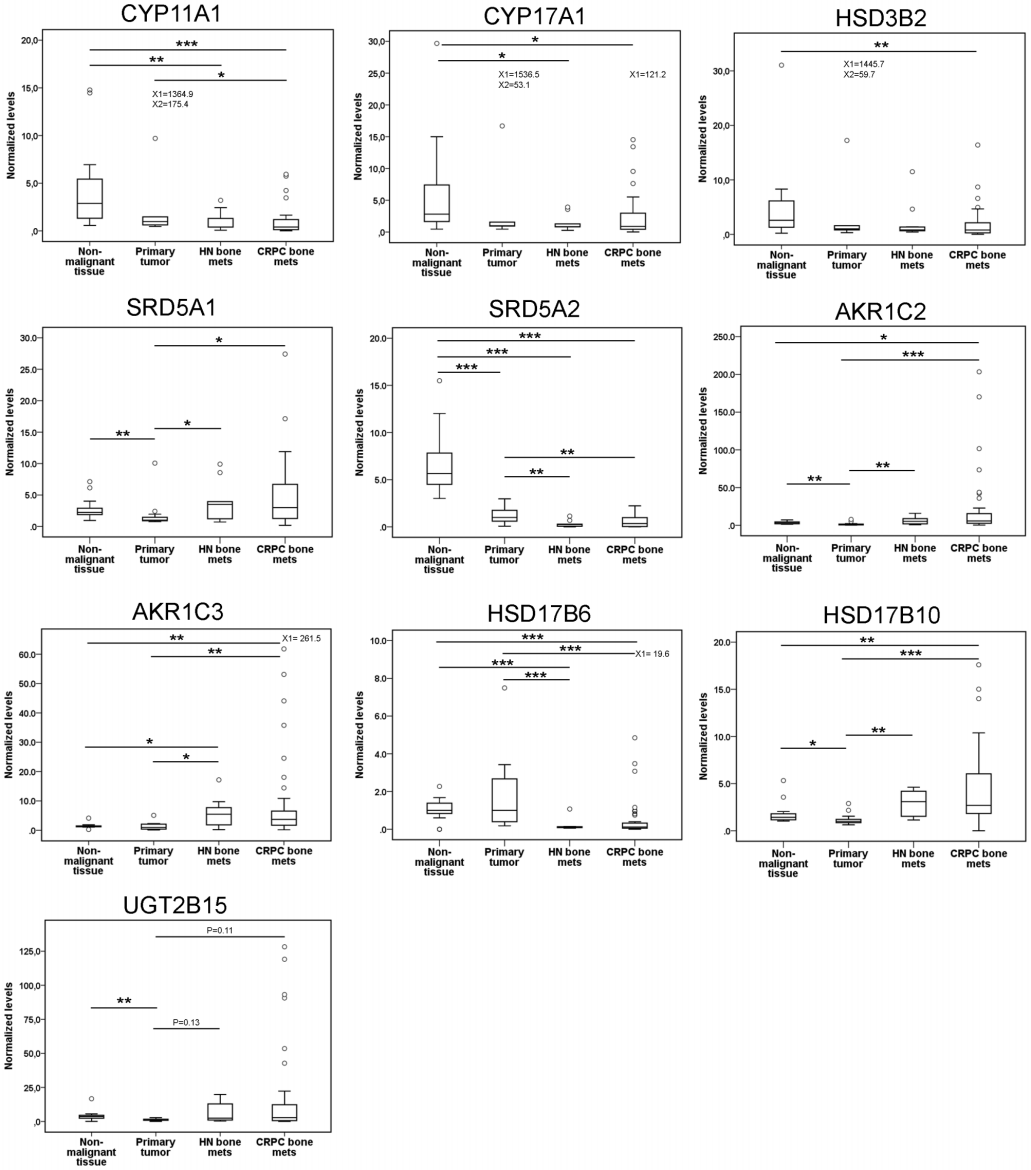
Relative mRNA levels of key enzymes involved in steroid metabolism in non-malignant prostate tissue (n = 13), primary prostate tumor tissue (n = 13), and in hormone-naïve (HN, n = 9) and castration-resistant prostate cancer (CRPC, n = 45) bone metastases when quantified with real-time RT-PCR. All mRNA levels were corrected for mRNA levels of the housekeeping gene RPL13A and normalized to the median value of the primary tumor samples. *P<0.05. ** P<0.01, *** P<0.001. Open circles indicate outliers and extremes. X indicates extreme levels outside the scale.

None of the analyzed enzymes in the steroidogenesis pathway (Figure 1 and 2) showed significantly different mRNA expression levels between HN and CRPC bone metastases (for comparison between metastases and prostate tissue see below). The variability between different CRPC metastases was however large and individuals with particularly high expression of specific steroidogenic enzymes were observed in the CRPC group (Figure 2).

### Prostate cancer bone metastases may convert weak circulating androgens into more potent ones but probably have low general capacity for de-novo steroidogenesis

Transcript levels of enzymes in the early steps of steroidogenesis were reduced in HN and CRPC bone metastases as compared to non-malignant prostate tissue (Figure 2); median CYP11A1, CYP17A1, and HSD3B2 mRNA levels were 0.15, 0.29, and 0.32 times in HN and 0.14, 0.32, and 0.31 times in CRPC bone metastases compared to the levels in non-malignant prostate tissue (P = 0.004, 0.025 and 0.071, and P = 0.00004, 0.019 and 0.01, respectively). The median CYP11A1 mRNA level in the CRPC metastases was 0.41 times compared to the level in the primary tumor tissue (P = 0.011, figure 2), while the CYP17A1 and HSD3B2 mRNA levels in CRPC bone metastases were not significantly different from levels in the primary tumor tissue. However, the CYP11A1 mRNA levels were correlated to the corresponding CYP17A1, HSD3B2, SRD5A1 and SRD5A2 mRNA levels in the CRPC metastases (Rs = 0.80, P<0.000001, Rs = 0.740, P<0.000001, Rs = 0.53, P<0.0002, and Rs = 0.70, P<0.000001), indicating that the early steps of steroidogenesis might be active in individual metastases.

Transcript levels of many enzymes involved in later steps of androgen synthesis were increased in metastases compared to non-malignant prostate and primary tumor tissue (Figure 2). The median AKR1C3 and AKR1C2 mRNA levels were 5.5 and 5.4 times higher in HN (P = 0.03, P = 0.007) and 3.7 and 5.8 times higher in CRPC bone metastases (P = 0.0006, P = 0.00008), respectively, as compared to primary prostate tumors. We observed a shift from SRD5A2 to SRD5A1 mRNA expression with progression from non-malignant prostate to PC and PC metastasis (Figure 2), which is in accordance with results in previous studies [14], [15], [19]. A similar shift from primarily HSD17B6 expression in non-malignant and primary prostate tumor tissue to mainly HSD17B10 expression in PC metastases was furthermore noted (Figure 2). The AKR1C3 mRNA levels were significantly correlated to the AKR1C2 and UGT2B15 mRNA levels (Rs = 0.39, P = 0.008 and Rs = 0.49, P = 0.001).

Taken together those results indicate that PC bone metastases in patients may have induced capacity to convert androgens with low AR affinity into more potent androgens, while *de novo* synthesis of androgens from cholesterol is less likely.

### Some bone metastases express very high levels of AKR1C3

Although none of the examined steroidogenic enzymes showed significantly increased expression levels in CRPC compared to HN bone metastases, individual CRPC metastases were noted to express very high transcript levels (Figure 2). One enzyme showing particularly high mRNA levels in a subgroup of CRPC metastases was AKR1C3. As this enzyme has the capacity to convert circulating DHEA and androstenedione (synthetized in the adrenals) into 5-androstenediol and testosterone, it was considered to be of particular interest.

**Figure 3:**
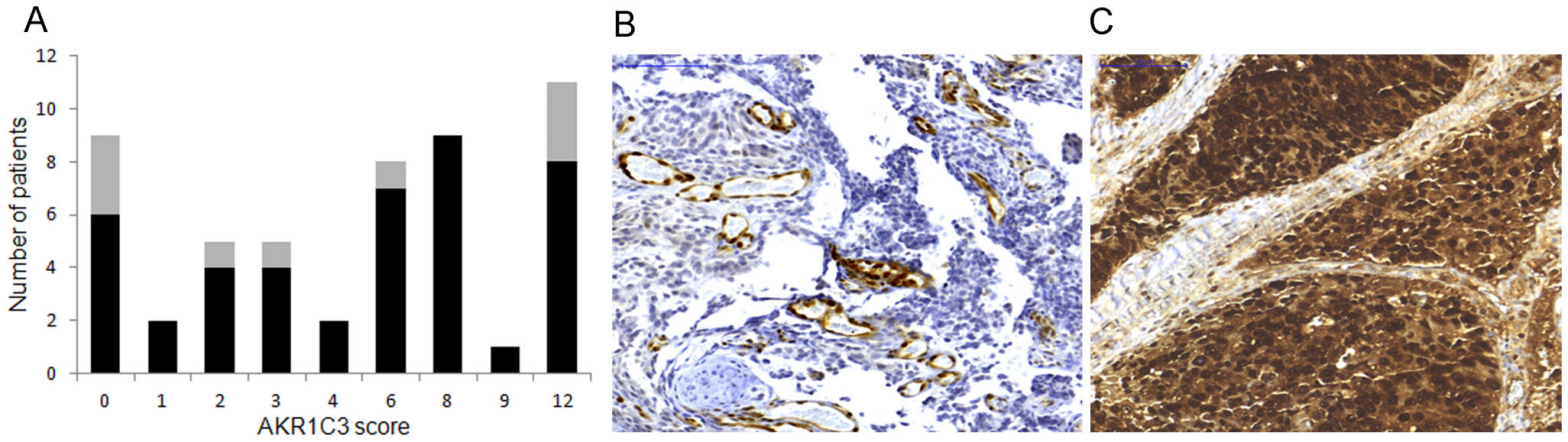
Immunohistochemical staining of AKR1C3 in bone metastases. A total score (ranging from 0–12) was obtained by multiplying the staining intensity score (0–3) in the tumor epithelial cells with the positive fraction score (1–4). (a) Histogram according to total AKR1C3 staining score in hormone-naïve (gray) and castration-resistant (black) bone metastases. (b) Representative section of bone metastasis with AKR1C3 total score 0. (c) Representative section of bone metastasis with AKR1C3 total score 12. Bar indicates 100 µM.

To determine whether the AKR1C3 mRNA levels in the metastases were reflected in the corresponding protein levels, we assessed the AKR1C3 protein expression by immunohistochemistry and by using a scoring system that took both staining intensity and distribution into account (Figure 3). Cytoplasmic AKR1C3 immunostaining was demonstrated in most tumor epithelial cells while nuclear staining was heterogeneously observed and not scored. All metastases showed strong positive staining in the endothelial cells, whereas variable staining was observed in fibroblasts and hematopoietic cells in the bone marrow. The AKR1C3 immunostaining total score was highly correlated to the AKR1C3 mRNA levels in the corresponding metastases (Rs = 0.73, P<0.000001, n = 51). High AKR1C3 expression was not due to AKR1C3 gene amplification, according to AKR1C3 gene copy number analysis of 6 metastases samples (3 samples with extreme AKR1C3 mRNA levels and total immunostaining scores of 12 and 3 samples with low AKR1C3 expression) in relation to 2 non-malignant prostate samples (data not shown).

In the CRPC bone metastases, the AKR1C3 total staining score was correlated to the UGT2B15 mRNA levels (Rs = 0.39, P = 0.009, n = 43), which might indicate DHT production in metastases with high AKR1C3 protein expression. It was however obvious that high AKR1C3 protein expression was not directly linked to high AR activity in CRPC metastases; 14 out of 18 (78%) samples with high AKR1C3 staining scores, defined as an AKR1C3 immunostaining score above median (6), and 15/25 (60%) samples with low AKR1C3 staining scores (≤6) showed high nuclear AR staining scores (≥8, above median, as defined in [5], P = 0.22). In accordance with previous results [5], high AR immunostaining in the metastases was associated with shorter cancer-specific survival after metastasis surgery (log-rank = 8.1, P = 0.004, n = 45). In contrast to this, there was no correlation found between the AKR1C3 immunostaining and cancer-specific survival of CRPC patients after metastasis surgery (data not shown). There was also no association observed between the AKR1C3 immunostaining score and the PSA staining score (data not shown) or the tumor cell proliferation index (Ki67 staining score, data not shown).

### Expression of steroidogenic enzymes in relation to levels of androgen receptor splice variants

**Figure 4:**
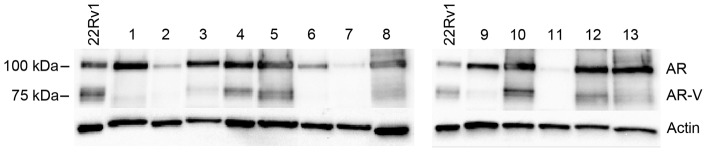
Western blot analysis of 13 patient samples showing representative expression patterns of the full length AR (110 kDa) and of LDB-truncated AR variants (AR-Vs) of about 80 kDa in CRPC bone metastases. Analysis was performed using an antibody targeting the N-terminal domain of the AR. The 22Rv1 cell extract was included as a positive control.

The AR protein levels were studied by immunoblot analysis of 29 CRPC bone metastases selected based on AR immunohistochemistry results to include AR low to AR high score metastases, and where adequate tissue remained to allow immunoblot analysis. The full length AR of approximate 110 kDa was detected in most cases while truncated AR-Vs of approximately 80 kDa, hypothesized to represent AR splice variants lacking the LBD C-terminal domain, were detected in some cases (Figure 4). The intensity of the 80 kDa-band was strongly correlated to corresponding AR-V7 mRNA level (Rs = 0.76, P<0.000002, data not shown), in accordance with own previous results [8]. In Hörnberg et al [8] patients with high metastasis levels of AR-V7 mRNA, defined to have mRNA levels in the upper quartile, were found to have extremely poor prognosis. Based on this knowledge, and using a similar approach, 7 samples analysed by immunoblotting were defined to express high levels of AR-Vs as the ratio between the intensities of the 80 and the 110 kDa bands were found within the 75^th^ percentile of the data (≥0.5).

**Figure 5:**
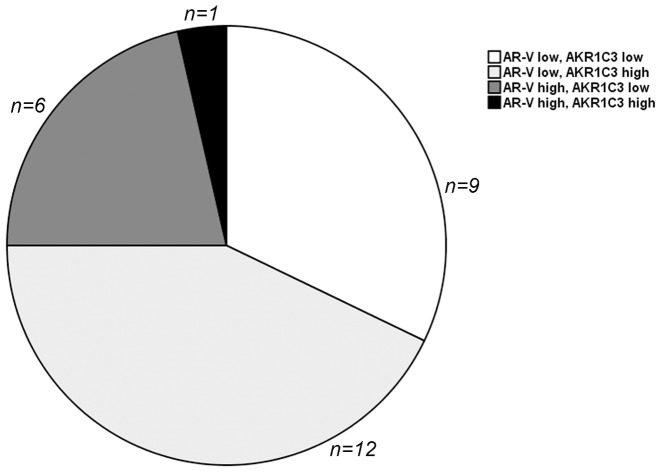
Characterization of 28 CRPC bone metastases according to protein levels of the steroidogenic enzyme AKR1C3 (defined as high if the AKR1C3 immunostaining score was above median, >6, and low if the score was ≤6) and protein levels of LBD-truncated AR variants (AR-Vs; defined as high if the ratio between the truncated 80 kDa and the full length 110 kDa AR bands was ≥0.5, quartile 4, and low if the ratio was below 0.5, quartile 1–3). High levels AKR1C3 were basically found in metastases with low levels of AR-Vs and vice versa (P = 0.049, according to Chi-square test).

Interestingly, only one out of 13 (7.8%) CRPC metastases with high AKR1C3 expression (immunostaining score above median) also showed high AR-V protein levels. This was a significantly lower fraction than found among metastases with low AKR1C3 levels (6/15, 40%, P = 0.049). We further hypothesized that tumor cell expression of AKR1C3 and constitutively active AR variants could be of relevance for individual tumor response to 2^nd^ line ADT and clinical progression [8], and the 28 bone metastases evaluated for both AKR1C3 and AR-V expression were therefore divided according to their AR-V and AKR1C3 protein expression levels, as shown in Figure 5, in order to demonstrate sub-groups of possible clinical relevance.

Notably, none of the metastases with homogeneous AKR1C3 staining, i.e fraction of positively stained cells above 75%, showed high AR-V levels compared to 7/15 metastases with <75% positively stained cells (P = 0.004). This observation deserves to be further examined, as it might indicate that high AR-V levels are induced preferable in tumors cells without endogenous steroidogenesis. Unfortunately, we were not able to study this hypothesized heterogeneity among tumor cells as antibodies for immunohistochemical evaluation of AR-Vs were not available.

## Discussion

Intra-tumoral synthesis of testosterone and DHT has been suggested to contribute to CRPC growth. In this study we compared expression levels of steroidogenic enzymes in CRPC bone metastases with levels in metastases obtained from un-treated patients and, surprisingly, found no induction of the examined enzymes in CRPC compared to HN metastases. We however found high expression levels of certain steroidogenic enzymes; SRD5A1, AKR1C2, AKR1C3, and HSD17B10, in bone metastases when compared to non-malignant prostate and primary prostate tumor tissue. Furthermore, we were able to show distinct differences in expression levels of AKR1C3 and levels of AR splice variants in individual cases of CRPC bone metastases, which we hypothesize could be of relevance for patient response to 2^nd^ line therapies.

Tissue androgens might be derived by *de novo* synthesis from cholesterol [20], [21] or by conversion of adrenal precursors into more potent steroids [22]. Our results showing generally high levels of AKR1C3 and SRD5A1 in metastases are in line with previous reports [14], [15], [16] and may indicate synthesis of testosterone and DHT in one and two steps, respectively, from adrenal-derived androstenedione. By the activity of SRD5A1, DHT could also be synthesized via 5α-reduction of androstenedione to androstanedione and then by further reduction of androstanedione to DHT by AKR1C3. This route for DHT synthesis, which bypasses testosterone, was recently shown by Chang and co-workers to dominate in CRPC cell lines as well as in patient metastases specimens stimulated with androstenedione [23]. Additionally, the high levels of AKR1C2 and HSD17B10 make DHT synthesis via androsterone and androstanediol possible in individual cases, similar to what has been observed in PC cell lines as well as in castration-resistant CWR22R xenografts in mice [24]. Intra-tumoral synthesis of androsterone and androstanediol could stimulate tumor growth not only as intermediate steroids in DHT synthesis, but also as potent AR activators in cases with mutated ARs [25]. Likewise could androstenediol, presumingly produced from DHEA in metastases with AKR1C3 activity, activate ARs with specific mutations [26]. In contrast to early reports [14], [15], but in line with Hofland and co-workers [16], we found low expression levels of CYP11A1, CYP17A1 and HSD3B2 in both untreated and CRPC metastases. High CYP11A1, CYP17A1 and HSD3B2 mRNA levels were instead found in non-malignant prostate tissue, probably due to high synthesis of those enzymes in normal prostate stromal cells, and the same was true for SRD5A2 and HSD17B6 [27], [28], [29], [30]. From this we would like to conclude that adrenal-derived androgens probably contribute to growth of CRPC bone metastases by their intra-metastatic conversion into more potent androgens, while *de novo* synthesis of androgens from cholesterol within metastases is less likely, except maybe in individual cases with correlated expression of CYP11A1, CYP17A1, and HSD3B2.

We found high expression levels of AKR1C3 in a sub-group of PC bone metastases, which confirm results previously reported in castration-resistant primary prostate tumors [16], [31] and CRPC tissue of different metastatic origin [14], [15], [32]. A rise in AKR1C3 activity could obviously contribute to the conversion of adrenal-derived steroids into more potent AR ligands, as discussed above, and to AR activation in CRPC. Likewise, Hamid and co-workers demonstrated AKR1C3 dependent synthesis of testosterone in CRPC cells stimulated with DHEA [31]. Beneficial effects of high AKR1C3 levels in HN metastases or in metastases with low nuclear AR immunostaining, as demonstrated for some cases in this study, are less clear, but may depend on other reactions driven by this enzyme. In addition to 17β-hydroxysteroid dehydrogenase (17β-HSD) activity AKR1C3 also possesses prostaglandin F (PGF) synthase activity which would lead to proliferation in PC cells independent of androgen and AR status due to formation of PGF epimers and activation of the FP receptor and also by the prevention of PGJ2 formation and its pro-differentiating and anti-proliferative effects [33].

In conclusion, we want to emphasize that increased tumor expression of steroidogenic enzymes in individual patients is not clearly associated with CRPC but merely associated with advanced tumor stage, as it can be seen not only in CRPC tissue of different origin [14], [15], [16], [31], [32] but also in previously un-treated, HN, bone metastases (this study). Furthermore, this study together with previous studies indicate that CRPC tumors could be classified according to distinct expression patterns of steroidogenic enzymes, and thus probably different mechanisms behind castration-resistance (this study and [16], [32], [34]). We additionally show that the sub-group of CRPC bone metastases expressing high levels of AKR1C3 is largely distinct from the sub-group expressing high levels of LBD-truncated, constitutively active AR-Vs. The possible clinical relevance of this is that patients with high AKR1C3 expression and low AR-V expression might be patients showing good response to treatment with abiraterone acetate (Cyp17 inhibition, [35]) and/or would benefit from drugs targeting AKR1C3 [36], while patients with high expression of constitutively active AR-Vs probably will not respond to abiraterone acetate or to any therapy targeting androgen synthesis or the LBD of the AR.

A limitation to this work is the restricted number of bone metastases, and furthermore the single metastasis sample examined from each patients, which obviously make conclusions regarding this heterogeneous disease and overall patient status uncertain. We are also aware that expression levels not necessarily reflect biological activities and our hypothesis that tumor expression pattern of steroidogenic AKR1C3 and constitutively active AR variants would predict response to 2^nd^ line therapies of CRPC needs to be tested upfront in clinical studies.

## Supporting Information

Table S1 **Primer sequences used for real-time RT-PCR analysis.**
(DOC)
