# Supplementary material for: Characterization of Prostate Cancer Bone Metastases According to Expression Levels of Steroidogenic Enzymes and Androgen Receptor Splice Variants
Source: PLoS One. 2013 Nov 7;8(11):e77407. doi: 10.1371/journal.pone.0077407 (PMC3820691; doi:10.1371/journal.pone.0077407)
Supplement: Table S1 — Primer sequences used for real-time RT-PCR analysis. (DOC) [file pone.0077407.s001.doc]

| **Table S1.** Primer sequences used for real-time RT-PCR analysis. | | | |
| --- | --- | --- | --- |
| **Gene** | **Amplicon** | **Primers 5´- 3´** | **References** |
|  | **size (bp)** |  |  |
| CYP11A1 | 83 | **CTGCATCTTCAGTCGTCTGTCC** | **15** |
|  |  | **GGTGACCACTGAGAACCCATTC** |  |
| CYP17A1 | 119 | **TCCCCAAGGTGGTCTTTCTGA** | **15** |
|  |  | **GTGGACAGGGGCTGTGAGTTAC** |  |
| HSD3B2 | 356 | **GGGCCCAACTCCTACAAGGA** |  |
|  |  | **ACTTGGGGCCTTCTTGGGGT** |  |
| SRD5A1 | 96 | **CCTGTTGAATGCTTCATGACTTG** | **15** |
|  |  | **TAAGGCAAAGCAATGCCAGATG** |  |
| SRD5A2 | 86 | **CTCTCTAAGGAAGGGGCCGAAC** | **15** |
|  |  | **GACAATGCATTCCGCAAACATA** |  |
| AKR1C2 | 268 | **CCTAAAAGTAAAGCTCTAGAGGCCGT** | **15** |
|  |  | **GAAAATGAATAAGATAGAGGTCAACATAG** |  |
| AKR1C3 | 85 | GGATAAGGCCTCCCTCGATT |  |
|  |  | GCGGAACCCAGCTTCTATTG |  |
| HSD17B10 | 160 | **GGCATGACACTGCCCATTG** | **a** |
|  |  | **GGTCACCCAGTCGGCTAGG** |  |
| UGT2B15 | 102 | **CGTTGTGCACATGTACCCTAAAA** | **15** |
|  |  | **CCATGTTCACATTTTCCTTCCTG** |  |
| AR-V7 | 125 | CCATCTTGTCGTCTTCGGAAATGTTATGAA | 9 |
|  |  | GCTTTGAATGAGGCAAGTCAGCCTTTCT |  |
| RPL13A | 125 | **GTACGCTGTGAAGGCATCAA** |  |
|  |  | **GTTGGTGTTCATCCGCTTG** |  |
| HSD17B6 | 84 | Hs00366258_m1 | Applied Biosystems |

# ^a^ [**Pfeiffer MJ**](http://www.ncbi.nlm.nih.gov/pubmed?term=Pfeiffer MJ%5BAuthor%5D&cauthor=true&cauthor_uid=21365123), [**Smit FP**](http://www.ncbi.nlm.nih.gov/pubmed?term=Smit FP%5BAuthor%5D&cauthor=true&cauthor_uid=21365123), [**Sedelaar JP**](http://www.ncbi.nlm.nih.gov/pubmed?term=Sedelaar JP%5BAuthor%5D&cauthor=true&cauthor_uid=21365123), [**Schalken JA**](http://www.ncbi.nlm.nih.gov/pubmed?term=Schalken JA%5BAuthor%5D&cauthor=true&cauthor_uid=21365123). (2011) Steroidogenic enzymes and stem cell markers are upregulated during androgen deprivation in prostate cancer. Mol Med 17(7-8):657-664.
